# Beyond the Usual: Breast, Pituitary and Gastric Metastases from Clear Cell Renal Cell Carcinomas—A Case Series with Review of Literature

**DOI:** 10.3390/diagnostics16121773

**Published:** 2026-06-09

**Authors:** Yin Ping Wong, Nur Liyana Khairuddin, Jegan Thanabalan, Geok Chin Tan

**Affiliations:** 1 Department of Pathology, Faculty of Medicine, Universiti Kebangsaan Malaysia, Kuala Lumpur 56000, Malaysia; cherishsasic1110@gmail.com; 2 Department of Surgery, Faculty of Medicine, Universiti Kebangsaan Malaysia, Kuala Lumpur 56000, Malaysia; jegan.dr@gmail.com

**Keywords:** clear cell, renal cell carcinoma, metastasis, breast, pituitary gland, stomach, gastric polyp, unusual sites, case series

## Abstract

**Background and Clinical Significance**: Clear cell renal cell carcinoma (ccRCC) is notorious for its aggressiveness and great propensity to metastasize to virtually any organ, with a dismal five-year survival rate. While metastases from ccRCC typically occur in the lungs, lymph nodes, bones and liver, involvement of atypical locations such as the breast, pituitary gland and stomach is extremely rare. These unusual metastases can masquerade as primary tumours at their respective sites, posing significant diagnostic challenges. **Case Presentation:** Here, we describe three cases of metastatic ccRCC to unusual anatomical sites following nephrectomy: (1) a patient who presented with a suspicious left-sided breast mass and synchronous liver and lung metastases six months following the initial diagnosis of ccRCC; (2) a patient who presented with diplopia, found to have a pituitary lesion four months after nephrectomy; and (3) a patient with known pre-existing lung metastases who developed upper gastrointestinal bleeding one year post-nephrectomy, in whom oesophagogastroduodenoscopy (OGDS) revealed an 8 mm pedunculated gastric polyp. Histopathological examination following biopsies of these lesions showed compact nests and sheets of malignant cells with clear to eosinophilic cytoplasm and distinct membranes. Immunohistochemically, these malignant cells demonstrated CD10 immunopositivity, and were negative for CK7 and CK20, in keeping with the diagnosis of metastatic ccRCC. **Conclusions**: This case series illustrates the rare metastatic behaviour of ccRCC with its potential to spread to uncommon sites. Awareness of such presentations is crucial, particularly in patients with a known history of ccRCC, as these lesions may clinically and radiologically mimic primary tumours of the affected sites. Careful evaluation of its histomorphological features and judicious use of immunohistochemical panels, together with clinical and radiological correlations, is the key to arriving at an accurate diagnosis.

## 1. Introduction

Renal cell carcinoma (RCC) is the most common primary malignancy of the kidney, accounting for approximately 2–3% of all adult cancers, with clear cell RCC (ccRCC) being the predominant histological subtype (comprising up to 70–80% of cases) [1]. The incidence of RCC is on the rise globally, largely attributed to the expansion of cross-sectional imaging and advanced imaging techniques, allowing for the incidental detection of renal masses at an earlier stage [2]. RCC is notorious for its highly aggressive biological behaviour due to its high propensity for early, often unpredictable metastatic disease at the time of initial diagnosis [3]. Approximately 30% of patients present with metastasis at diagnosis, while up to 40% of those with localised disease subsequently develop distant metastasis following nephrectomy performed with curative intent [4].

The classical sites of RCC metastases are lung (70%), lymph node (45%), bone (32%), liver (18%), adrenal gland (10%) and brain (8%) [5]. In contrast, metastasis to atypical sites such as pituitary, gastrointestinal tract, breast, thyroid, pancreas, skin, salivary gland and muscle is exceedingly rare, yet has been previously documented [5]. Importantly, these metastatic lesions frequently mimic primary tumours of the involved organs both clinically and radiologically, posing significant diagnostic challenges and potentially leading to misdiagnosis and inappropriate clinical management.

Here, we report three cases of metastatic RCC involving these three exceptional sites—the breast, pituitary gland and stomach—highlighting the clinicopathological features, immunohistochemical workup and diagnostic difficulties we encountered in each. We also provide a review of the recent literature from the last 10 years published in English on previously reported cases of metastatic RCC in unusual sites, with particular focus on these three anatomical locations, i.e., the breast [6,7,8,9,10,11,12,13,14,15,16,17,18,19,20,21,22,23,24,25,26,27,28], pituitary [29,30,31,32,33,34,35,36,37,38,39,40] and stomach [41,42,43,44,45,46,47,48,49,50,51,52,53,54,55,56,57,58,59,60,61,62,63,64,65,66,67,68,69,70,71,72,73,74,75,76,77,78,79] (Supplementary Table S1).

## 2. Case Presentations

### 2.1. Case 1: Breast Metastasis

A patient with a known history of right-sided ccRCC presented to the breast clinic with a palpable left breast lump, six months following curative right nephrectomy. As the initial surgery was performed at an external institution, the baseline staging computed tomography (CT) scan records from the time of primary diagnosis were unavailable. Histopathological examination of the nephrectomy specimen revealed renal vein thrombosis. The patient noticed that the mass had been gradually increasing in size over the preceding three months. There was no family history of breast carcinoma.

Mammography revealed a large heterogeneous enhancing mass occupying the upper and lower quadrant of the left breast measuring 71 × 36 × 63 mm (AP × W × CC), with a poor fat plane with the left pectoralis muscle. There were also a few subcentimetre contrast-enhancing axillary lymph nodes noted. The breast lesion was provisionally categorised as a BI-RADS 5 lesion. A core needle biopsy of the breast lesion was subsequently performed.

**Figure 1:**
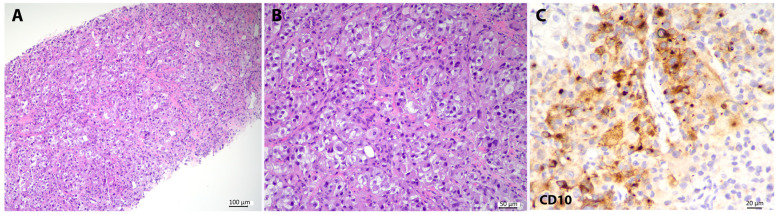
Histopathological examination of the breast mass. (**A**) Diffuse infiltration by malignant cells in infiltrating nest, separated by delicate, thin-walled sinusoidal vasculature (Haematoxylin & Eosin (H&E), ×200). (**B**) The malignant cells display pleomorphic hyperchromatic nuclei with occasional conspicuous nucleoli and abundant clear to eosinophilic cytoplasm (H&E, ×400). (**C**) The malignant cells show CD10 immunopositivity (CD10, ×400).

Histological examination of the breast biopsy demonstrated infiltrating nests and sheets of malignant cells set within a delicate, thin-walled sinusoidal vasculature. The malignant cells displayed mildly pleomorphic and hyperchromatic nuclei, some with conspicuous eosinophilic nucleoli visible at 100× magnification, with abundant clear to vacuolated and eosinophilic cytoplasm. Mitoses were readily seen. No in situ carcinoma component was identified. Immunohistochemistry demonstrated positivity for CD10 and vimentin, with negativity for CK7, CK20, oestrogen receptor (ER), progesterone receptor (PR), and HER2. The morphological and immunophenotypical features were consistent with metastatic ccRCC to the breast (Figure 1).

Staging CT of the chest, abdomen and pelvis demonstrated multiple mass lesions seen in the liver and lung, consistent with distant metastases. The patient’s condition deteriorated in the ward postoperatively, and the patient subsequently succumbed to the disease prior to referral to the oncology team for further management.

### 2.2. Case 2: Pituitary Metastasis

A patient with a history of right-sided ccRCC, treated by radical nephrectomy four months prior, presented with progressive diplopia and tunnel vision, which deteriorated to complete blindness of the left eye. The initial staging CT of the chest, abdomen and pelvis prior to nephrectomy was negative for distant metastasis. Endocrinological assessment demonstrated biochemical evidence of panhypopituitarism, including secondary hypothyroidism and hypogonadism. There was no clinical evidence of diabetes insipidus at presentation.

Magnetic resonance imaging (MRI) of the brain demonstrated a heterogeneously enhancing sellar mass measuring 19 × 20 × 35 mm, with suprasellar extension. The differential diagnoses included a pituitary macroadenoma and metastatic disease, given the patient’s known history of ccRCC. The patient subsequently underwent transsphenoidal resection of the sellar mass. Intraoperatively, the tumour was noted to be adhered to the optic nerve and optic chiasm. Adequate decompression, however, was successfully achieved with preservation of both structures.

**Figure 2:**
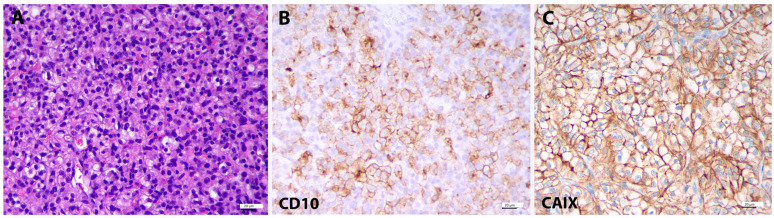
Histopathological examination of the sellar mass. (**A**) Infiltrating malignant cells in nests and alveolar clusters, separated by delicate, richly branching sinusoidal vasculature (H&E, ×400). (**B**) The malignant cells demonstrate CD10 (CD10, ×400) and (**C**) CAIX immunopositivity (CAIX, ×400).

Histopathological examination of the resected tissue revealed nests and alveolar clusters of polygonal tumour cells with clear to eosinophilic cytoplasm and centrally placed nuclei. They were separated by a delicate, richly branching sinusoidal vasculature. Immunohistochemistry studies showed that the tumour cells were immunoreactive for CD10, CAIX and vimentin, and were negative for synaptophysin and chromogranin A, thereby excluding a pituitary adenoma. CK7 and CK20 were also negative (Figure 2). A diagnosis of metastatic ccRCC to the pituitary gland was rendered.

Postoperative computed tomography (CT) of the brain demonstrated a residual enhancing lesion within the sellar region measuring 22 × 18 × 18 mm. The patient subsequently received adjuvant stereotactic radiotherapy to the pituitary fossa, delivered at a total dose of 20 Gy in five fractions over one week. Systemic targeted therapy with pazopanib was also initiated. At one-year post-operative follow-up, the patient remained clinically well, with a repeat CT of the brain demonstrating a stable residual pituitary mass with no sign of disease progression. The patient was subsequently defaulted on follow-up.

### 2.3. Case 3: Gastric Metastasis

A patient with a background history of left-sided ccRCC with synchronous lung metastases, treated by radical nephrectomy one year earlier, presented with a one-week history of melena and symptomatic iron deficiency anaemia (Haemoglobin level 7.2 g/dL). There was no prior history of peptic ulcer disease or use of non-steroidal anti-inflammatory drugs.

**Figure 3:**
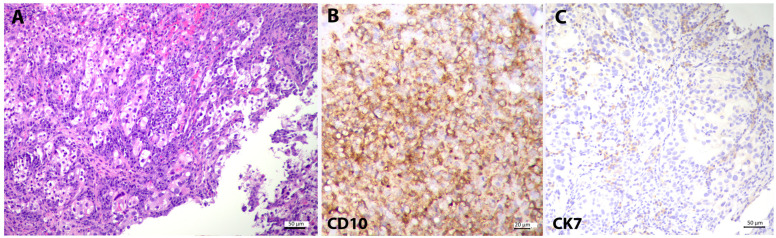
Histopathological examination of the gastric polyp. (**A**) Infiltrating malignant cells in solid nests, interspersed with a delicate, arborising capillary network (H&E, ×200). (**B**) The malignant cells demonstrate CD10 immunopositivity (CD10, ×400) and (**C**) CK7 immunonegativity (CK7, ×400).

Oesophagogastroduodenoscopy (OGDS) identified a single 8 mm pedunculated polyp in the body of the stomach. Snare polypectomy was performed. Microscopic examination revealed infiltration of the lamina propria by tumour cells arranged in solid nests, interspersed with a delicate arborising capillary network. The tumour cells exhibited round to oval nuclei with finely granular open chromatin, inconspicuous nucleoli and abundant clear cytoplasm. Immunohistochemically, the tumour cells demonstrated diffuse immunopositivity for pancytokeratin (CKAE1/AE3) and CD10, while being negative for CK7 and CK20 (Figure 3).

The overall histomorphological and immunophenotypic features were consistent with metastatic ccRCC. Referral to the oncology team for consideration of systemic therapy was planned; however, the patient’s condition deteriorated, and the patient subsequently succumbed to the disease.

## 3. Discussion

Renal cell carcinoma is the 14th most common malignancy and the 16th leading cause of cancer-related death worldwide, with an estimated 434,840 new cases diagnosed in a year. It accounts for approximately 2–3% of all adult cancers [80]. The incidence is about twice as high in men compared to women. Notably, higher incidence rates are observed in developed regions including North America and Europe, while lower incidence is seen in Asia and Africa. In Malaysia, the age-standardised incidence rate (ASR) is relatively low at 2.7 cases per 100,000 population per year. However, despite this low incidence, outcomes appear less favourable, with a relatively high mortality-to-incidence ratio (MIR 0.52 in 2018), suggesting later-stage presentation and potential disparities in access to early diagnosis and treatment [81].

Among the many recognised risk factors for RCC, hypertension, insulin resistance/diabetes mellitus, obesity, cigarette smoking and chronic kidney disease are the most strongly and consistently associated with RCC development. In addition to these modifiable risk factors, hereditary factors play a significant role in determining genetic predisposition to RCC. Several genes have been implicated in RCC pathogenesis and hereditary RCC syndromes, including *VHL*, *ELOC*, *TSC1/2*, *MET*, *FLCN*, *FH*, *SMARCB1*, *BAP1*, *CHEK2* and *PTEN* [82]. These genetic alterations serve as the linchpin in cancer initiation and progression.

Von Hippel–Lindau syndrome (VHLS) is a rare, but highly penetrant autosomal dominant hereditary neoplastic disorder caused by germline mutations in the VHL tumour suppressor gene, with an estimated incidence of about 1 in 36,000 births [83]. It was first described through the work of Eugen von Hippel and Arvid Lindau in the early 20th century. The VHL gene, located on the short arm of chromosome 3 (3p25), encodes a protein that forms part of an E3 ubiquitin ligase complex responsible for targeting hypoxia-inducible factors (HIFs), especially HIF-1α, which plays a key role in the cellular response to hypoxia. Notably, loss of at least one copy of chromosome 3p is observed in over 90% of sporadic ccRCCs [84]. VHL inactivation in (both sporadic and hereditary) ccRCCs leads to stabilisation and accumulation of HIF-1α irrespective of the oxygen status. This results in constitutive activation of downstream genes involved in angiogenesis (e.g., VEGF), metabolism, and cell survival. This highly vascular tumour microenvironment not only drives tumourigenesis but also facilitates tumour progression and subsequent dissemination [84].

RCC demonstrates a spectrum of gross appearances corresponding with its histological subtype. Classically, ccRCC exhibits a golden-yellow cut surface due to its high lipid content, often accompanied by areas of haemorrhage, necrosis, cystic degeneration and occasional calcification. In contrast, papillary RCC typically appears brown to haemorrhagic and granular, while chromophobe RCC often shows a mahogany-brown appearance. Most RCCs are well-circumscribed and cortical-based [85].

Histologically, RCC encompasses several subtypes with distinct morphological characteristics. ccRCC, the most common pathological subtype, accounts for 70–80% of cases. It is composed of nests and alveolar arrangement of neoplastic cells with abundant clear cytoplasm due to intracytoplasmic lipid and glycogen, separated by a delicate network of thin-walled vasculature. Papillary RCC, comprises 7–14% of tumours, is characterised by papillary or tubulopapillary architecture lined by neoplastic cells with eosinophilic or basophilic cytoplasm, often accompanied by foamy macrophages and psammoma bodies. Chromophobe RCC, representing approximately 2–4% of RCCs, shows large polygonal neoplastic cells with pale to eosinophilic cytoplasm, prominent cell borders and perinuclear halos. Several histopathological features such as high nuclear grade, tumour necrosis and lymphovascular invasion are associated with more aggressive behaviour. Furthermore, sarcomatoid and rhabdoid differentiation represent high-grade transformation and confer an adverse prognosis [85].

Tumour invasion is essential for metastatic spread, but not all invasive tumours metastasize. RCC demonstrates a greater propensity for haematogenous spread, facilitated by its rich sinusoidal vasculature and frequent early invasion of the renal vein [86,87]. The so-called tumour thrombi (TT) in the renal vein, reported in approximately 15% of cases, provide a direct conduit for tumour cells to access the systemic circulation. This vascular route of spread partly explains the diverse and sometimes unpredictable metastatic patterns observed in RCC.

Mechanistically, the progression of metastasis is driven by dysregulation of key oncogenic signalling pathways, including phosphoinositide 3-kinase (PI3K)/AKT/mammalian target of rapamycin (mTOR), Ras/MAPK (mitogen-activated protein kinase) and Wnt/β-catenin pathways. These oncogenic pathways promote tumour cell proliferation, survival and migratory cues, besides enabling tumour cells to withstand adverse microenvironments and to establish secondary growth at distant sites [88]. In RCC metastasis, these pathways work synergistically to induce epithelial–mesenchymal transition (EMT), enabling tumour cells to detach, invade surrounding stroma and enter the circulation. Concurrent extracellular matrix (ECM) remodelling, mediated by matrix metalloproteinases, further facilitates invasion and metastatic seeding [87].

Successful metastasis of cancer cells depends on the ability of circulating tumour cells to adapt to and to colonise distant organ microenvironments. RCC exhibits a unique form of organotropism, reflected by its capacity to metastasize to nearly any site in the body, including rare locations such as the pituitary gland, breast and stomach. Based on the existing literature, this behaviour is thought to be mediated by tumour–stroma interactions, chemokine signalling and exosome-mediated intercellular communication. For instance, CXCR4/CXCL12 axis plays a pivotal role in directing tumour cell migration and homing to specific metastatic niches [89], while tumour-derived exosomes contribute by conditioning pre-metastatic niches through modulation of the local immune milieu, extracellular matrix remodelling and promotion of a pro-tumourigenic environment that facilitates tumour cell engraftment [90]. The exact molecular underpinnings dictating why ccRCC cells home to rare sites remain incompletely elucidated, requiring further mapping of these unique organotropic pathways.

Accurate diagnosis of metastatic RCC relies on a high index of suspicion and meticulous histopathological evaluation, especially when it presents at unusual sites. Morphologically, metastatic RCC often recapitulates the features of the primary tumour, with ccRCC demonstrating nests or alveolar arrangements of tumour cells with abundant clear to eosinophilic cytoplasm and a delicate capillary network [85]. In metastatic sites such as the breast, stomach and pituitary gland, these lesions may closely mimic primary neoplasms native to the organ, making recognition of subtle architectural and cytoplasmic features critical.

Histological overlap with primary tumours at metastatic sites poses a significant diagnostic challenge. For instance, metastatic ccRCC to the breast may resemble primary breast carcinomas with clear cell or apocrine features [11], while gastric involvement may mimic poorly differentiated adenocarcinoma or gastrointestinal stromal tumours [88]. Pituitary metastases may be mistaken for primary pituitary neuroendocrine tumours, especially when exhibiting nested or solid growth [91]. Additional features such as rich sinusoidal vasculature, intratumoral haemorrhage and the absence of in situ components (e.g., lack of ductal carcinoma in situ in breast lesions) may provide important diagnostic clues favouring metastasis.

Immunohistochemistry plays a pivotal role in confirming renal origin in metastatic lesions. PAX8 is the most sensitive and widely used marker, supporting renal epithelial differentiation and serving as a key discriminator from most primary tumours at these sites [92]. CcRCC typically demonstrates diffuse membranous CAIX expression with a characteristic “box-like” pattern, along with CD10 and vimentin positivity. In contrast, CK7 is usually negative or only focally positive in ccRCC, aiding distinction from primary breast and gastric adenocarcinomas, which are typically CK7 positive. For papillary and chromophobe RCC metastases, CK7 and CD117 may be contributory [92].

Judicious use of site-specific immunohistochemical markers cannot be overemphasised to exclude a primary tumour at the metastatic site. For instance, in breast lesions, the absence of immunomarkers such as GATA3, mammaglobin and gross cystic disease fluid protein 15 (GCDFP-15) supports a non-mammary origin and helps steer the diagnosis away from primary breast carcinoma [93]. Similarly, in gastric lesions, lack of CK20 and CDX2 expression argues against a primary gastrointestinal adenocarcinoma, especially when the morphology is not classic for a gastric primary. In the pituitary, immunonegativity for neuroendocrine markers such as synaptophysin and chromogranin further supports a metastatic disease rather than primary pituitary neoplasm [94], especially in lesions lacking the usual adenohypophyseal differentiation. Notably, a panel-based immunohistochemical approach remains essential, as no one single marker is entirely specific.

Careful correlation with clinical history remains indispensable, particularly in patients with a history of prior RCC and the often strikingly long disease-free interval before metastatic presentation.

This case series has several limitations that should be acknowledged. First, the retrospective, single-centre design and small sample size preclude drawing definitive conclusions regarding the clinical behaviour of ccRCC. Furthermore, our cases reflect heterogeneous follow-up and incomplete outcome data; specifically, the patients in Cases 1 and 3 succumbed to their disease shortly after diagnosis, while the patient in Case 2 was lost to long-term follow-up. As these cases were sourced from archived pathology records, there is inherent selection bias. Additionally, the immunohistochemical panels performed were variable across the cases, limiting direct comparability. Notably, PAX8 staining—considered the most sensitive marker for renal epithelial origin—was not performed due to unavailability of the antibody at the time of diagnosis. However, the combination of classic histomorphology and alternative immunohistochemical markers adequately supported the final diagnoses in these patients.

## 4. Conclusions

Metastatic ccRCC to the breast, pituitary gland, and stomach is exceedingly rare. Awareness of such presentations is crucial, particularly in patients with a known history of ccRCC, as these lesions may clinically and radiologically mimic primary tumours. This case series serves as a reminder of the importance of considering metastatic disease in unusual locations, and highlights the pivotal role of careful histopathological evaluation and judicious use of immunohistochemistry panels in arriving at an accurate diagnosis.

## Data Availability

The original contributions presented in the study are included in the article/Supplementary Material, further inquiries can be directed to the corresponding author.

## Supplementary Materials

The following supporting information can be downloaded at https://www.mdpi.com/article/10.3390/diagnostics16121773/s1, Table S1: Summary of Previous Reported Cases (from Year 2016–2026) of ccRCC Metastasis to Atypical Sites, Pertaining to Stomach, Pituitary Gland and Breast.
