# Supplementary material for: Beyond the Usual: Breast, Pituitary and Gastric Metastases from Clear Cell Renal Cell Carcinomas—A Case Series with Review of Literature
Source: Diagnostics (Basel). 2026 Jun 9;16(12):1773. doi: 10.3390/diagnostics16121773 (PMC13297709; doi:10.3390/diagnostics16121773)
Supplement: Supplementary file 1 [file diagnostics-16-01773-s001.zip › diagnostics-4326090-supplementary.pdf]

SUPPLEMENTARY TABLE S1:
Summary of Previous Reported Cases (from Year 2016 – 2026) of ccRCC Metastasis to Atypical Sites, Pertaining to Stomach, Pituitary Gland and Breast.

Breast

Author (Year)	Age (Year) /Gender	Clinical Presentation	Tumour Size	Other Metastatic Sites	Time Interval to Metastasis (Year)	Initial treatment	Treatment after metastasis	Outcomes
Arjunan et al. (2016) [6]	45/F	Right breast lump	2.9x2.8cm	Axillary lymph nodes	0	Right radical nephrectomy	Right modified radical mastectomy; AC & 5FU	Alive
Koch et al. (2016) [7]	55/F	Left breast lump	9mm	Liver	8	Left nephrectomy	Lumpectomy & ablation	Alive
Amadu et al. (2017) [8]	75/F	Left breast lump	NA	No	4	Right radical nephrectomy	NA	NA
Ishigaki et al. (2017) [9]	82/F	Incidental CT findings	1.3cm	No	9	Left nephrectomy	Partial mastectomy	Alive
Dhannoon et al. (2017) [10]	63/F	Incidental mammographic findings	4x4cm	No	5	Right radical nephrectomy	WLE	Alive
Xu et al. (2017) [11]	68/F	Left breast lump	1.0x0.6cm	No	10	Nephrectomy	WLE	Alive
Ikarashi et al. (2018) [12]	57/F	Incidental CT findings	0.8cm	Pancreas	2	Right radical nephrectomy	Breast segmental resection (Metastasectomy)	Alive
Tandon et al. (2018) [13]	79/F	Left breast lump	3x3cm	No	1	Left nephrectomy	WLE	Alive
Tandon et al. (2018) [13]	83/F	Right breast lump	1x1cm	No	3	Nephrectomy	WLE	Alive

Kitahara et al. (2018) [14]	71/F	Left breast lump	0.6x0.7x0.4cm	Lung, right axillary lymph nodes	9	Bilateral nephrectomy	Refuse pharmacotherapy	Alive
Parihar et al. (2018) [15]	36/F	Left breast lump	NA	Thyroid, lung, lymph nodes	0	NA	NA	NA
Nguyen et al. (2020) [16]	57/F	Incidental mammographic findings	0.9x0.8x0.6cm	No	NA	Left radical nephrectomy	Sunitinib	Alive
Aleman-Cabrera et al. (2021) [17]	51/F	Right breast lump	2x2cm	Right thigh	10	Left radical nephrectomy	Breast conserving surgery	NA
Verma et al. (2021) [18]	60/F	Right breast lump	2.5x2.7cm	No	0	Cytoreduction nephrectomy	Radiotherapy	LTFU
Ali et al. (2021) [19]	73/F	Right breast lump	2.0x1.3cm	Pancreas, liver, brain	3	Left nephrectomy	Sunitinib	NA
Khurram et al. (2021) [20]	65/F	Right breast lump	6.3x5.6cm	Lung, cerebellum	0	No	No	Succumbed (immediately after Dx)
Elouarith et al. (2022) [21]	69/F	Left breast lump	2.5cm	NA	18	Right nephrectomy	Lumpectomy	NA
Spasic et al. (2023) [22]	82/F	Right breast lump	2cm	No	11	Right radical nephrectomy	Metastasectomy	Alive
Pernicone & Fabrega-Foster (2023) [23]	76/F	Incidental mammographic findings	2.5cm	Lung, skeletal muscle, subcutaneous tissue	0	No	Pembrolizumab, lenvatinib	Alive
Sahoo et al. (2025) [24]	54/F	Incidental CT findings	0.5cm	No	1	Radical nephrectomy	WLE	NA

Dai et al. (2025) [25]	67/F	Progressive jaundice, weight loss	2cm	Pancreas	21	Left radical nephrectomy	Lumpectomy & pancreaticoduodenectomy	Succumbed (8 months after Dx)
Aslan et al. (2025) [26]	45/F	Bilateral breast lump	NA	Lung, mediastinum	0.6	Right radical nephrectomy	Everolimus, sunitinib	Succumbed (13 months after Dx)
Xiao et al. (2026) [27]	86/F	Left breast lump	2x1.5cm	Lung, brain	25	Radical nephrectomy	Lumpectomy	NA
Wang et al. (2026) [28]	54/M	Right breast lump	NA	Lung, pancreas, chest wall, retroperitoneum, iliac vessels	0	Partial nephrectomy	Sunitinib	Alive
Current case	NA	Left breast lump	7.1x3.6x6.3cm	Liver, lung	0.5	Right radical nephrectomy	No	Succumbed (a few days after Dx)

Abbreviations: AC & 5FU – Adriyamycin, cyclophosphamide & 5-fluorouracil; CT – computed tomography; Dx – diagnosis; F – female; LTFU – lost to follow up; M – male; NA – data not available; WLE – wide local excision

Pituitary

**Author (Year)**	**Age (Year) /Gender**	**Clinical Presentation**	**Metastatic Sites**	**Other Metastatic Sites**	**Time Interval to Metastasis (Year)**	**Initial treatment**	**Treatment after metastasis**	**Outcomes**
Ravnik et al. (2016) [29]	54/F	Burning eye pain, double vision, headache	Pituitary	No	6	Nephrectomy	Decompressive pituitary tumour surgery, radio- and chemotherapy	Alive
Payandeh et al. (2016) [30]	50/M	Polyuria, polydipsia	Pituitary	Lung, bone	0	Right nephrectomy	Bevacizumab, sunitinib	Alive

Wendel et al. (2016) [31]	61/M	Deteriorated visual acuity, bitemporal hemianopia	Pituitary	No	2	Right radical nephrectomy	Palliative brain irradiation	Succumbed (30 months after Dx)
Di Nunno et al. (2018) [32]	45/M	Incidental MRI brain findings	Pituitary	Chest, pancreatic lymph nodes, cerebellum	14	Right radical nephrectomy	Gamma knife surgery, sunitinib	Alive
Selby et al. (2018) [33]	62/M	Decrease visual acuity	Pituitary	Gastric fundus, bone, hilum, choroidal	21	Left radical nephrectomy	Transsphenoidal resection of pituitary mass, nivolumab	Succumbed (5 months after Dx)
Gandhi et al. (2020) [34]	70/M	Weakness, diplopia, headache	Pituitary	Adrenal, retrocaval lymph nodes	0	Right radical nephrectomy	Transsphenoidal resection of pituitary mass, stereotactic radiotherapy to pituitary	Alive
Moon et al. (2021) [35]	69/F	Headache, cerebellar ataxia	Pituitary	No	15	Radical nephrectomy	Subtotal resection of pituitary mass, pazopanib	Alive
Li et al. (2021) [36]	58/M	Headache, deteriorate visual acuity	Pituitary	Subcutaneous tissue	5	Right radical nephrectomy	Transsphenoidal resection of pituitary mass	Alive
Oven et al. (2022) [37]	51/F	Visual disturbance, headache	Pituitary	No	0.4	Left radical nephrectomy	Surgical resection of pituitary mass, sunitinib	NA
Venkataramana et al. (2023) [38]	41/M	Diplopia, headache	Pituitary	NA	NA	NA	Total resection surgery	Alive
Mazar-Atabaki et al. (2023) [39]	53/F	Headache	Pituitary	No	0	Right radical nephrectomy	Sellar radiotherapy	NA

Avula et al. (2024) [40]	59/M	Olfactory hallucination, bitemporal vision loss	Pituitary	No	2	Right nephrectomy	Resection of sellar & suprasellar mass	Alive
Current case	NA	Diplopia, tunnel vision	Pituitary	No	0.3	Right radical nephrectomy	Transsphenoidal resection of pituitary mass, Pazopanib	Alive

Abbreviations: Dx – diagnosis; F – female; M – male; MRI – magnetic resonance imaging; NA – data not available

Stomach

**Author (Year)**	**Age (Year) /Gender**	**Clinical Presentation**	**Metastatic Sites**	**Other Metastatic Sites**	**Time Interval to Metastasis (Year)**	**Initial treatment**	**Treatment after metastasis**	**Outcomes**
Akay et al. (2016) [41]	72/M	Fatigue, loss of appetite	Gastric fundus	No	20	Left radical nephrectomy	Refuse chemotherapy	NA
Al Juboori et al. (2017) [42]	67/M	Dyspepsia, anaemia	Gastric body	Lung, bone	13	Right radical nephrectomy	Everolimus	Succumbed (10 months after Dx)
Uehara et al. (2017) [43]	73/M	Incidental CT findings	Gastric fundus	Lung	6	Cytoreduction nephrectomy	EMR, Sunitinib	Alive
O’Reilly et al. (2017) [44]	59/F	Incidental CT findings	Gastric fundus	Peritoneal	1.25	Left radical nephrectomy	Sunitinib	NA
Grosser & He Huang (2018) [45]	70/M	Upper GI bleed	Stomach	Pancreas	4	NA	NA	LTFU
Hemmerich et al. (2018) [46]	84/M	Dysphagia, GERD	Gastric body	No	5	NA	NA	NA

Hemmerich et al. (2018) [46]	58/M	GERD	Gastric fundus	Adrenal, pancreas, liver, lung	6	NA	NA	NA
Hemmerich et al. (2018) [46]	74/M	Upper GI bleed	Gastric antrum	No	NA	NA	NA	NA
Hemmerich et al. (2018) [46]	72/F	GERD, anaemia	Gastric body	Liver, pancreas	NA	NA	NA	NA
Hemmerich et al. (2018) [46]	66/M	Melena, anaemia	Gastric body	Liver, pancreas, mesentery, brain	10	NA	NA	NA
Kiyani et al. (2018) [47]	77/M	Dyspepsia	Gastric body	No	20	Nephrectomy	Polypectomy, Sunitinib	NA
Arakawa et al. (2018) [48]	80/F	Anorexia	Gastric greater curvature	Liver, lung	0	NA	Axitinib	NA
Mubarak et al. (2018) [49]	83/F	Melena, anaemia	Gastric lesser curvature	No	0	NA	Polypectomy and clipping, refuse further therapy	LTFU
Weissman et al. (2019) [50]	85/M	Dyspepsia, malaise	Proximal stomach	No	2	Right nephrectomy	Palliative chemotherapy	NA
Weissman et al. (2019) [50]	70/M	Malaise, anaemia, weight loss	Gastric fundus	No	0	NA	Palliative chemotherapy	NA
Kinoshita et al. (2019) [51]	60/M	Routine GI endoscopy	Gastric body	Gallbladder	3	Right nephrectomy	Gastric wedge resection	Alive
Mehta et al. (2020) [52]	63/M	Postprandial abdominal pain	Stomach	Pancreas, duodenum	NA	NA	NA	NA
Yoshida et al. (2020) [53]	85/F	Anaemia, upper GI bleed	Gastric body	Pancreas	15	Right radical nephrectomy	EMR	Alive

Bernshteyn et al. (2020) [54]	68/M	Melena	Gastric body	Lung, skin, rectum	NA	Right total nephrectomy	NA	NA
Parmar et al. (2021) [55]	65/M	Incidental CT findings	Gastric fundus	No	0	Right radical nephrectomy	Excision, Pazopanib	Alive
Prudhomme et al. (2021) [56]	69/M	Incidental CT findings	Gastric lesser curvature	No	8	Right partial nephrectomy	Gastric wedge resection	Alive
Hakim et al. (2021) [57]	86/F	Melena, haematemesis	Gastric body	Lung, mediastinal lymph nodes, bone	10	Left nephrectomy	Hot snare polypectomy with radiation	Alive
Koterazawa et al. (2021) [58]	70/F	Progressive weight loss	Gastric body	No	0	Right radical nephrectomy	ESD	Alive
Degerli et al. (2022) [59]	73/M	Upper GI bleed	Fundic gland polyp	Lung, mediastinal lymph nodes, right rib	14	Right radical nephrectomy	First line: Sunitinib	Alive
Kim et al. (2022) [60]	70/F	Incidental CT findings	Gastric body	Opposite kidney, bone	7	Left nephrectomy	Cryoablation	NA
Chen et al. (2022) [61]	65/M	Incidental medical check up	Gastric fundus	No	5	Radical nephrectomy	ESD, Sunitinib, then Axitinib	Alive
McIlwaine et al. (2022) [62]	80/F	Malaise, weight loss	Gastric greater curvature	Lung	21	Left radical nephrectomy	Refuse further therapy	LTFU
Tapasak et al. (2022) [63]	77/M	Upper GI bleed	Gastric cardia	Diaphragm	9	Left radical nephrectomy	Total gastrectomy	NA
Cerrella Cano et al. (2023) [64]	77/M	Asthenia, dizziness, upper GI bleed	Gastric greater curvature	Rib, abdominal wall	22	Left radical nephrectomy	Atypical gastrectomy of greater curvature	Alive

Saifi et al. (2023) [65]	65/M	Incidental MRI findings	Gastric fundus and body	Lung, peritoneal	8	Right nephrectomy	NA	NA
Yamashita et al. (2024) [66]	83/M	Melena, dizziness	Gastric body	Lung	6	Left radical nephrectomy	Partial gastrectomy	Alive
Yan et al. (2024) [67]	82/M	Epigastric pain, melena	Gastric body	Opposite kidney, pancreas, spleen	12	Left radical nephrectomy	ESD, Sorafenib	Alive
Alomari et al. (2024) [68]	76/M	Melena, dizziness, fatigue	Gastric body	No	11	Left nephrectomy	ESD	Alive
Mori et al. (2024) [69]	50/M	Incidental CT findings	Gastric body	Pancreas	11	Left nephrectomy	Pyloric gastrectomy, total pancreatectomy	Alive
Sabete-Ortega et al. (2024) [70]	84/F	Melena, anaemia	Gastric body	No	9	Right radical nephrectomy	EMR	Alive
Peregoy et al. (2024) [71]	49/M	Suspected acute pancreatitis	Gastric body	Pancreas	NA	NA	Cabozantinib	NA
Nario et al. (2024) [72]	73/F	Melena, lethargy	Gastric body	Lung, bone	11	Left nephrectomy	Polypectomy, Axitinib	Succumbed after LTFU
Magara et al. (2024) [73]	60/F	Heartburn	Gastric greater curvature	Pancreas	12	Left nephrectomy	Gastric wedge resection & pancreaticoduodenal resection	Alive
Goncalves et al. (2025) [74]	83/M	Melena, epigastric discomfort, vomiting	Gastric cardia	No	30	Nephrectomy	Palliative radiotherapy	Alive
Yao et al. (2025) [75]	62/M	Epigastric discomfort	Gastric fundus	Lung	1	Left nephrectomy	Axitinib, denosumab, toripalimab	Alive

Iwamuro et al. (2025) [76]	58/M	Epigastric discomfort	Gastric body	Pancreas	0	NA	Sunitinib, then everolimus, then temsirolimus	Succumbed (10 months after Dx)
Fukami et al. (2026) [77]	62/M	Incidental OGDS findings	Gastric greater curvature	Pancreas	4	Right partial nephrectomy	ESD, panopanib	Alive
Nikolaus & Sigdel (2026) [78]	65/M	Nausea, vomiting, abdominal distension	Gastric body	Lung, liver, brain	0	NA	Pembrolizumab, lenvatinib	Alive
Wang et al. (2026) [79]	68/M	Abdominal pain, diarrhoea	Gastric body	No	0	Right radical nephrectomy	No	Alive
Current case	NA	Melena, anaemia	Gastric body	Lung	1	Left radical nephrectomy	No	Succumbed (a few days after Dx)

Abbreviations: CT – computed topography; Dx – diagnosis; EMR – Endoscopic mucosal resection; ESD – Endoscopic submucosal resection; F – female; GERD – gastro-oesophago reflux disease; GI – gastrointestinal, LTFU – lost to follow up; M – male; NA – data not available/not mentioned; OGDS – oesophago-gastroduodenoscopy
